# Functionality in metal–organic framework minerals: proton conductivity, stability and potential for polymorphism†
†Electronic supplementary information (ESI) available. CCDC 1868681–1868685. For ESI and crystallographic data in CIF or other electronic format see DOI: 10.1039/c8sc05088k


**DOI:** 10.1039/c8sc05088k

**Published:** 2019-04-02

**Authors:** Igor Huskić, Novendra Novendra, Dae-Woon Lim, Filip Topić, Hatem M. Titi, Igor V. Pekov, Sergey V. Krivovichev, Alexandra Navrotsky, Hiroshi Kitagawa, Tomislav Friščić

**Affiliations:** a Department of Chemistry , McGill University , Montreal , Canada . Email: tomislav.friscic@mcgill.ca; b Peter A. Rock Thermochemistry Laboratory and NEAT ORU , University of California Davis , Davis , CA , USA . Email: anavrotsky@ucdavis.edu; c Division of Chemistry , Graduate School of Science , Kyoto University , Kitashirakawa-Oiwakecho, Sakyo-ku , Kyoto , 606-8502 Japan . Email: kitagawa@kuchem.kyoto-u.ac.jp; d Kola Science Centre , Russian Academy of Sciences , Apatity and Department of Crystallography , Saint Petersburg State University , Saint Petersburg , Russia; e Faculty of Geology , Lomonosov Moscow State University , Moscow , Russia

## Abstract

Rare metal–organic framework (MOF) minerals stepanovite and zhemchuzhnikovite can exhibit properties comparable to known oxalate MOF proton conductors, including high proton conductivity over a range of relative humidities at 25 °C, and retention of the framework structure upon thermal dehydration. They also have high thermodynamic stability, with a pronounced stabilizing effect of substituting aluminium for iron, illustrating a simple design to access stable, highly proton-conductive MOFs without using complex organic ligands.

## Abstract 2

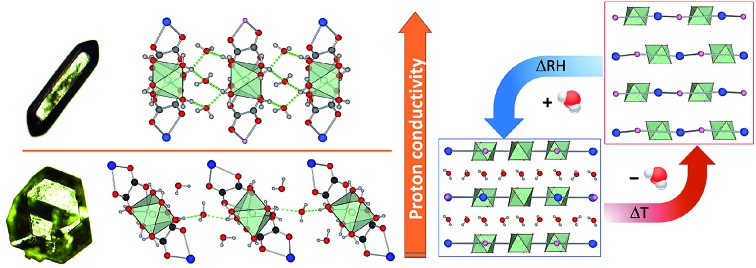
Metal–organic framework minerals stepanovite and zhemchuzhnikovite can exhibit high proton conduction and structure retention on dehydration.

## Introduction

**Fig. 1:**
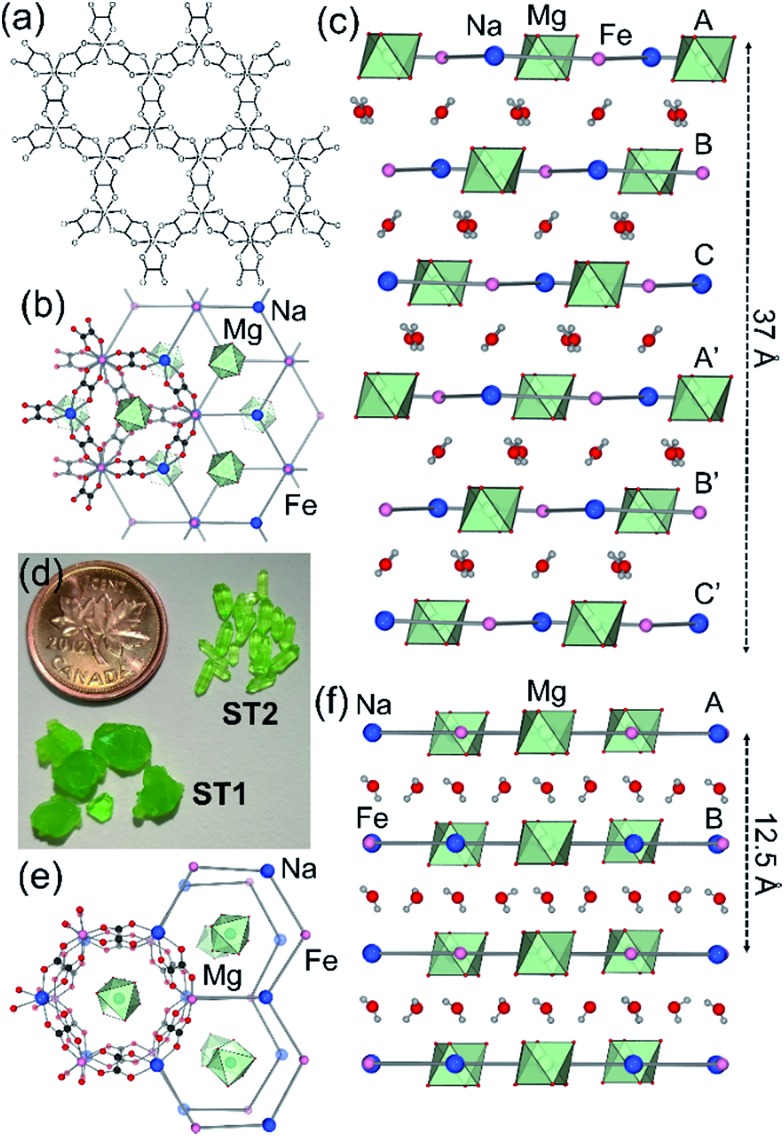
(a) Scheme of **hcb**-layers in stepanovite and zhemchuzhnikovite; (b) **hcb**-layers in synthetic stepanovite (**ST1**) viewed along and (c) perpendicular to the crystallographic *c*-axis, showing ABCA′B′C′ stacking. (d) Samples of **ST1** and **ST2** shown next to a Canada penny coin (∼1.9 cm diameter) for size comparison; (e) **hcb**-layers in **ST2** viewed parallel to the *c*-axis, showing channel formation and (f) perpendicular to the *c*-axis, showing AB stacking. Green octahedra represent coordination environments of Mg(H_2_O)_6_^2+^ ions.

Whereas most minerals are inorganic solids,^1^ recent work highlighted complex organic or metal–organic structures of minerals found in unusual or extreme environments.^2^ Examples include mellitic acid on Mars,^3^ hydrocarbon cocrystals as prospective minerals on Titan,^4^ metal oxalate coordination polymers (humboldtine, lindbergite),^5^ or geoporphyrins (abelsonite).^6^ The search to discover and understand the properties of such non-conventional minerals has been galvanized by the Carbon Mineral Challenge project,^7^ exploring the role of carbon in mineral and biological evolution on Earth and other planets.^8^ A recent addition to this set of unusual natural structures are metal–organic frameworks (MOFs) in the form of rare minerals zhemchuzhnikovite (**ZH**, [Mg(H_2_O)_6_][NaFe_*x*_Al_1–*x*_(C_2_O_4_)_3_]·3H_2_O, *x* ∼ 0.6) and stepanovite (**ST**, [Mg(H_2_O)_6_][NaFe(C_2_O_4_)_3_]·3H_2_O), discovered in the Lower Lena region of Siberia.^9^,^10^ Single crystal X-ray diffraction analysis of natural samples reveals that the minerals are based on open two-dimensional (2-D) honeycomb (3,6)-topology (**hcb**-topology) anionic nets composed of oxalate linkers and a combination of Na^+^ with either only Fe^3+^ (in **ST**) or a mixture of Al^3+^ and Fe^3+^ nodes (in **ZH**) (Fig. 1a).^11^ The sheets are separated by layers of ordered guest water molecules, and charge-balanced by Mg(H_2_O)_6_^2+^ ions located in cavities of each **hcb**-sheet. These structures are hybrid organic–inorganic materials analogous to oxalate MOFs investigated for optical, magnetic and particularly proton-conductive properties.^12^,^13^ The structural similarity raises the exciting possibility that **ZH** and **ST** minerals should exhibit functional properties expected for related MOFs, notably proton conductivity and stability upon heating or guest removal.^11^,^14^

However, scarcity of the minerals has prevented the measurement of their properties. Moreover, while it was reported that **ZH** and **ST** undergo reversible loss of guest water molecules,^11^ there has been no evidence that such dehydration proceeds with retention of the **hcb**-networks, which would be expected in a functional MOF material.^14^

We now show that **ZH** and **ST** can indeed exhibit functional behavior on par with known oxalate MOF proton conductors. Specifically, synthetic mineral analogues exhibit high proton conductivities at room temperature, undergo thermal removal of included water guests without changes to the underlying **hcb**-networks and, by using solution calorimetry, we demonstrate they are also of high thermodynamic stability.

## Experimental

All reagents were available commercially and were used without additional purification. Oxalic acid, NaOH, NaHCO_3_, MgO, MgCl_2_·6H_2_O and FeCl_3_·6H_2_O were obtained from Sigma Aldrich.

### Synthesis of **ST1** and **ST2**

Synthesis of **ST1** and **ST2** was performed by dissolving FeO(OH) (first obtained by mixing of aqueous solutions of FeCl_3_·6H_2_O and NaHCO_3_, followed by filtration) in an aqueous solution of oxalic acid. After addition of NaOH and MgCl_2_·6H_2_O, the solution was filtered and placed in a refrigerator at 4 °C for crystallization and slow evaporation. The resulting **ST1** and **ST2** crystals were separated by hand, based on crystal morphology. Slow evaporation led to mixtures containing **ST1** as the major product, while faster evaporation, *e.g.* in a rotary evaporator, gave **ST2** as the major product.

### Synthesis of **ZH**

Synthesis of **ZH** was performed in two steps. In the first step, NaMgAl(C_2_O_4_)_3_·9H_2_O was obtained by dissolving aluminum metal in an aqueous solution of oxalic acid with sonication, followed by addition of NaOH, MgCl_2_·6H_2_O and filtration. Evaporation of the liquid led to crystallization of NaMgAl(C_2_O_4_)_3_·9H_2_O, isolated by filtration. In the second step, equivalent amounts of NaMgAl(C_2_O_4_)_3_·9H_2_O and NaMgFe(C_2_O_4_)_3_·9H_2_O (synthesized as described for **ST1** and **ST2** above) were dissolved in a small amount of water. After several days at 4 °C, yellow-green needles of **ZH** were isolated. Bulk **ZH** sample was obtained by milling equimolar amounts of NaMgAl(C_2_O_4_)_3_·9H_2_O and NaMgFe(C_2_O_4_)_3_·9H_2_O with 40 μL H_2_O for 30 min in 10 mL Teflon® jars with one zirconia ball (10 mm diameter) at 25 Hz.

### Dehydration of **ST1** and **ST2** single crystals

Dehydration of **ST1** and **ST2** single crystals was performed with gentle heating at 50 °C under vacuum over a period of 12 hours. The compounds are further denoted as **ST1d** and **ST2d** denoting dehydrated **ST1** and **ST2** respectively.

### Powder X-ray diffraction (PXRD)

Powder X-ray diffraction (PXRD) data for rehydration experiments was collected on a PROTO AXRD benchtop instrument equipped with a DECTRIS MYTHEN2R 1D detector, using nickel-filtered CuK_α_ (*λ* = 0.154056 Å) radiation. Rehydration was performed in a custom-made sealed sample holder^15^ kept at 98% relative humidity with the aid of a saturated aqueous solution of K_2_SO_4_.

### Variable temperature (VT)

Variable temperature (VT) dehydration experiments and verification after impedance measurements were performed on a Bruker D8 Advance instrument equipped with a LYNXEYE XE-T detector using nickel-filtered CuK_α_ (*λ* = 0.154056 Å) radiation. The setup was equipped with Anton Paar CHC plus^+^ chamber. Diffractograms were collected in a stepwise fashion in a dry environment. After each collection (*ca.* 15 min), temperature in the chamber was raised by 1 °C at a rate of 1 °C min^–1^ and a new collection started.

### Crystal structures of **ST2**, **ST1d** and **ST2d**

Crystal structures of **ST2**, **ST1d** and **ST2d** were determined by single crystal X-ray diffraction using a Bruker D8 APEX2 X-ray diffractometer and graphite-monochromated MoKα (*λ* = 0.71073 Å) radiation. Structures were solved by intrinsic phasing in SHELXT^16^ and refined on F^2^ using SHELXL.^17^ Wherever possible, hydrogen atoms participating in hydrogen bonds were located from the electron density map. Crystallographic data in CIF format has been deposited with the Cambridge Structural Database (CSD), CCDC ; 1868681–1868685.

### Fourier-transform infrared attenuated total reflectance (FTIR-ATR)

Fourier-transform infrared attenuated total reflectance (FTIR-ATR) studies were done on a Bruker VERTEX 70 instrument with a PLATINUM diamond crystal ATR unit.

### Thermogravimetric analysis and differential scanning calorimetry (TGA and DSC) analysis

Thermogravimetric analysis and differential scanning calorimetry (TGA and DSC) analysis was done on a Mettler-Toledo TGA DSC 1 Star system thermobalance using alumina crucibles under a stream of nitrogen (50 mL min^–1^) and a heating rate of 5 °C min^–1^ from 25 °C until 200 °C, and in a stream of air (50 mL min^–1^) at a heating rate of 10 °C min^–1^ from 200 °C to 700 °C. Sample size was between 2 mg and 10 mg.

### Deuterated **ST1** and **ZH**

Deuterated **ST1** and **ZH** were prepared by first fully dehydrating the parent phases NaMgFe(C_2_O_4_)_3_·9H_2_O (**ST1**) and NaMgAl(C_2_O_4_)_3_·9H_2_O under vacuum at 70 °C, after which the materials were dissolved in D_2_O and recrystallized in a dry N_2_ atmosphere. Deuteration was confirmed by FTIR-ATR, and the identity of samples was verified by PXRD.

### Alternative current (AC) impedance measurement

Alternative current (AC) impedance measurement was performed by the conventional two-probe method. Samples (**ST1**, **ST2**, and **ZH1**) were pelletized about ∼1.2 mm in thickness and 2.5 mm in diameter. The pellet was connected to electrode with gold paste and gold wires (50 μm in diameter). The test is performed in a temperature- and humidity-controlled chamber, which was connected to a Solartron SI 1260 Impedance/Gain-Phase Analyzer and 1296 Dielectric Interface. The impedance measurement was executed in the frequency range from 1 Hz to 10 MHz at 298 K. Proton conductivity and activation energy barrier were estimated by eqn (1) and (2), where σ is the conductivity (S cm^–1^), *L* is the measured sample thickness (cm), *S* is the electrode area (cm^2^) and *Z* is the impedance value. In eqn (2), *A* is a pre-exponential factor, *k* is the Boltzmann constant, and *E*_a_ is the activation energy of ionic conduction.1
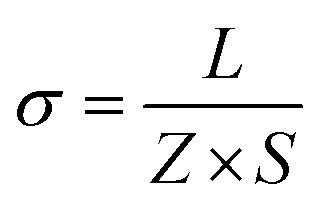

2
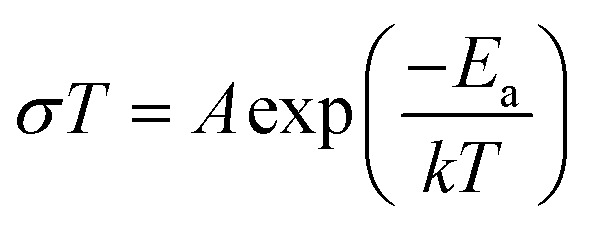



### Water vapor sorption measurements

Water vapor sorption measurements were carried out on **ZH**, **ST1**, and **ST2** using an automated micropore gas analyser BELSORP-max (MicrotracBEL Corp.). Before measurement, the water for adsorbate was degassed through a freeze-thaw cycle, and all samples were activated at 80 °C under vacuum (10^–3^ Pa) overnight. The water isotherms were measured at each equilibrium pressure by the static volumetric method at 298 K under *P*/*P*_0_ = 0.95.

### Calorimetric measurements

Calorimetric measurements to determine the enthalpies of dissolution (Δ*H*_ds_) of MOF samples (**ST1**, **ST2** and **ZH**) and starting materials were performed using a CSC (Calorimetry Sciences Corporation) 4400 isothermal microcalorimeter at 25 °C. Around 5 mg of the sample was hand-pressed to form a pellet and dropped to a Teflon cell in the calorimeter, filled with 25 g of 5 molar aqueous HCl solution. The solvent was isothermally equilibrated for at least 3 h under mechanical stirring before the introduction of the sample, and the sample was allowed to dissolve in the cell for at least 2 hours, ensuring the return of baseline back to its initial position. In each experiment, the solvent in the cell was replaced with new, fresh solvent.

The calibration of the instrument was performed using a NIST standard reference material KCl. The calibration was done by dissolving 15 mg of the KCl pellet into 25 g of H_2_O (type-1, resistivity = 18.2 MΩ cm), which corresponds to a reference concentration of 0.008 mol kg^–1^ at 25 °C. The calibration factor was obtained by correlating the integrated data with a known enthalpy of dissolution and dilution of 0.008 mol kg^–1^ KCl. For each sample, at least 4 measurements were performed. The uncertainties given in the result represents 95% confidence interval.^18^ Due to incomplete dissolution of γ-FeOOH in 5 molar HCl, its exact concentration was determined by inductively coupled plasma mass spectrometry (ICP-MS) using an Agilent 8900 ICP-MS. The supernatant after calorimetry measurement was diluted 10X and 100X with 3% HNO_3_ (by volume) prepared from concentrated Trace Metal Grade HNO_3_ (Fisher Scientific) and deionized water (resistivity value of 18.2 MΩ cm).

## Results and discussion

Synthetic **ZH** was prepared following our previous procedure (see ESI†).^11^ Analysis of synthetic **ZH** by PXRD was consistent with the reported mineral structure, based on **hcb**-layers stacked along the 12.6 Å crystallographic *c*-axis to form channels filled with Mg(H_2_O)_6_^2+^ ions. The mineral **ST** exhibits a different structure,^11^ in which **hcb**-layers stack in ABCA′B′C′ fashion, resulting in a longer crystallographic *c*-axis of ∼37 Å (Fig. 1b and c, where the A and A′ notations indicate layers with the same overall positions but rotated around the *c*-axis by 180° relative to each other).^9^–^11^ However, a crystallographic study of synthetic samples by Piro *et al.*^19^ suggested that **ST** is isostructural to **ZH**, *i.e.* exhibiting AB layer stacking along a 12.6 Å *c*-axis. To resolve this discrepancy, we have now systematically investigated crystallization of **ST** under a variety of conditions, unexpectedly revealing that slow evaporation from water produces large hexagonal plate-like crystals, whereas rapid evaporation produces elongated crystals with a hexagonal cross-section. Single crystal X-ray diffraction revealed that slowly grown plates (termed stepanovite 1, **ST1**) are isostructural to the mineral, with ABCA′B′C′ stacking of **hcb**-sheets and a ∼37 Å *c*-axis perpendicular to large hexagonal crystal faces (Table 1, entries 1, 2). In contrast, elongated crystals (termed stepanovite 2, **ST2**) exhibit structural features and crystallographic parameters (Table 1, entries 3, 4) similar to **ZH**, including AB-stacking of **hcb**-sheets and guest-filled channels parallel to a 12.4 Å *c*-axis (Fig. 1e and f). Identical results were observed using X-ray diffraction data collected at room temperature and at 100 K, confirming that the difference between structures of **ST1** and **ST2** is not related to temperature. The existence of **ST2**, a polymorph of synthetic stepanovite that is isostructural to zhemchuzhnikovite, offers an explanation of discrepancy between mineral structure and the synthetic sample structure reported by Piro *et al.*^19^

**Table 1:** Crystallographic parameters of synthetic stepanovite polymorphs **ST1**^*a*^ and **ST2** collected at 298 K and 100 K, and of corresponding dehydrated phases **ST1d** and **ST2d**

Entry	Sample	*T* (K)	*a* (Å)	*c* (Å)	Space group
1	**ST1** ^*a*^	298^*a*^	9.8367(13)^*a*^	36.902(5)^*a*^	*R*3*c*^*a*^
2	**ST1**	100	9.8670(9)	36.735(3)	*R*3*c*
3	**ST2**	298	17.0483(4)	12.4218(4)	*P*3*c*
4	**ST2**	100	17.0033(11)	12.4160(8)	*P*3*c*
5	**ST1d**	298	9.7745(2)	29.7940(11)	*R*3
6	**ST2d**	298	9.756(3)	10.117(3)	*P*3

^*a*^From ref. 11.

Stabilities of synthetic **ST1**, **ST2**, and **ZH** were investigated by room temperature acid dissolution calorimetry.^18^,^20^ The enthalpies of formation of synthetic minerals were calculated from herein measured dissolution enthalpies (Δ*H*_ds_) of the minerals and relevant starting materials (oxalic acid, binary oxides, hydroxides, and oxyhydroxides), and a properly designed thermodynamic cycle (see ESI† for details and equations). This enabled the evaluation of relative energetic stabilities (Table 2) of both **ST** polymorphs and **ZH**, as well as their enthalpies of formation from oxides (Δ*H*_f,ox_) and from the elements (Δ*H*_f,el_).

**Table 2:** Measured enthalpies of solution in 5 N HCl (Δ*H*_s_, kJ mol^–1^) and calculated enthalpies of formation from oxides Δ*H*_f,ox_ and elements Δ*H*_f,el_ of synthetic **ST1**, **ST2** and **ZH** (for the rest of the thermodynamic data see ESI)

Sample	Δ*H*_s_	Δ*H*_f,ox_	Δ*H*_f,el_
**ST1**	91.38 ± 0.66	–422.31 ± 2.29	–5847.41 ± 2.59
**ST2**	92.15 ± 0.47	–423.07 ± 2.24	–5848.19 ± 2.55
**ZH**	64.81 ± 1.06	–434.03 ± 3.20	–6113.98 ± 3.43

The very exothermic Δ*H*_f,ox_ indicates that the formation of **ST1**, **ST2** and **ZH** from binary oxides is thermodynamically driven. The results also show that the difference in energetic stability of **ST1** and **ST2** is within the experimental error of calorimetric measurements. However, **ZH** has a noticeably more exothermic enthalpy of formation from oxides compared to **ST** polymorphs, indicating that structure stabilization by substitution of iron by aluminum is far larger than the effect of polymorphism. This trend of greater stability relative to binary oxide components for the aluminum compounds relative to the iron ones has been seen in many inorganic systems containing both Al^3+^ and Fe^3+^, such as in spinels,^21^ jarosite–alunite and natrojarosite–natroalunite solid solutions^22^ and zoisite, clinozoisite solid solutions.^23^

Although **ST1** and **ST2** are very similar in enthalpy, there is some indication from different growth conditions that **ST2** is a kinetic polymorph, while **ST1** is thermodynamically preferred. This is supported by the observation that **ST2** converts to **ST1** by shaking or brief milling with small amounts of water. The metastable character of **ST2** compared to **ST1** would agree with Goldsmith's simplexity principle,^24^ stating that kinetically-favored phases are often structurally simpler than their thermodynamically stable counterparts.^25^ Our calculations of information-based structural complexity parameters^26^ show that **ST1** has a very complex structure (*I*_G_ = 5.618 bits/atom; *I*_G,total_ = 1617.979 bits/cell), whereas the structure of **ST2** is intermediate in complexity (*I*_G_ = 4.099 bits/atom; *I*_G,total_ = 393.510 bits/cell).

**Fig. 2:**
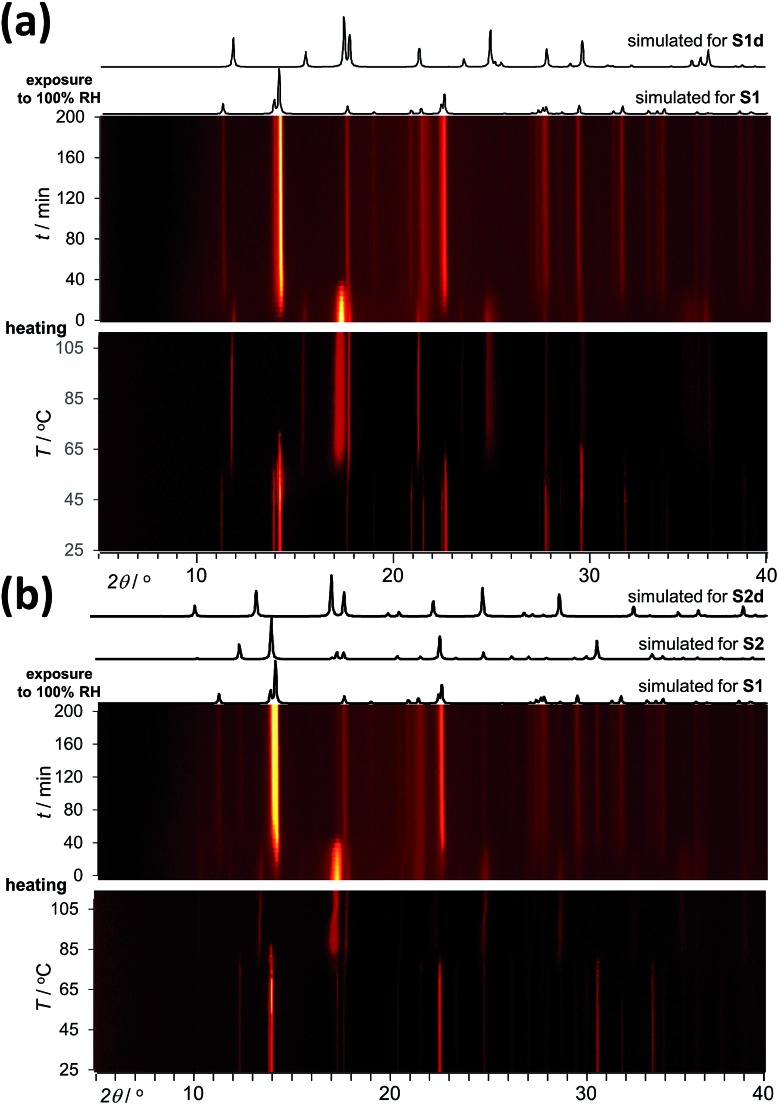
PXRD data for a powdered sample of (a) **ST1** and (b) **ST2** being (bottom) first dehydrated by heating (2 °C per hour, from 25 °C to 110 °C) and subsequently (top) exposed to 100% RH at room temperature. Simulated PXRD patterns for **ST1**, **ST2**, **ST1d** and **ST2d** are shown above the *in situ* data plots.

**Fig. 3:**
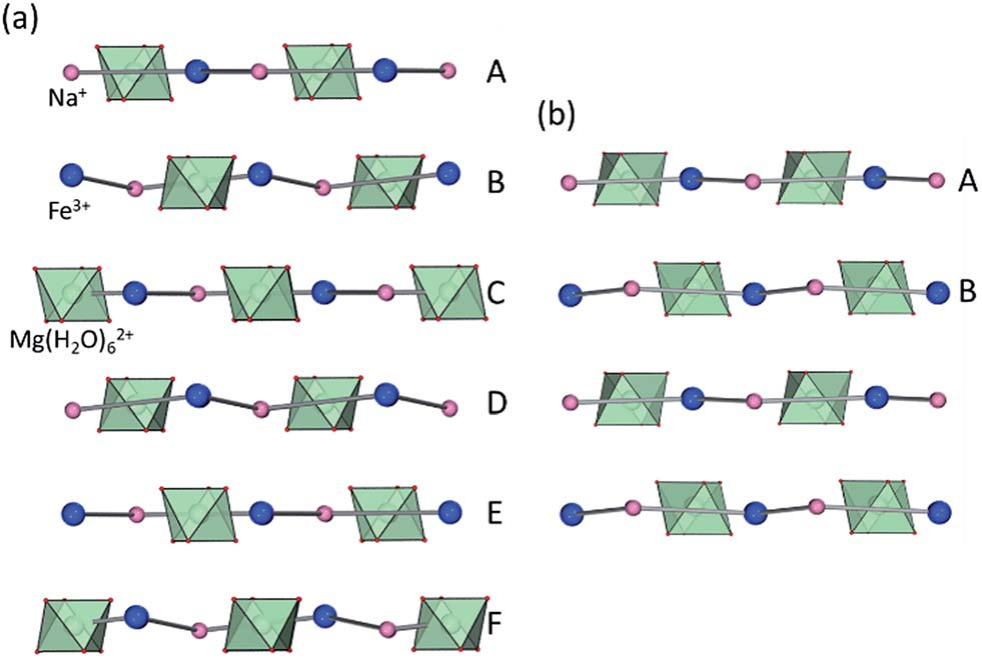
Arrangement of **hcb**-layers in (a) **ST1d** and (b) **ST2d**, viewed perpendicular to the crystallographic *c*-direction.

Analysis by TGA (see ESI†) and variable-temperature powder X-ray diffraction (VT-PXRD, Fig. 2a and b) show that **ST1** and **ST2** lose three equivalents of guest water molecules upon mild heating, forming new crystalline phases **ST1d** and **ST2d**, respectively. Furthermore, crystals of **ST1** and **ST2** retained their integrity upon dehydration, providing diffraction-quality crystals of **ST1d** and **ST2d**. Structure analysis shows that dehydration leads to partial (**ST1**) or complete (**ST2**) corrugation of **hcb**-layers (Fig. 3), which lowers the space group symmetry of **ST1d** and **ST2d** compared to the parent phases (Table 1, entries 5, 6). In **ST1d** the **hcb**-layers adopt a more complex ABCDEF repeat pattern as a result of the original ABCA′B′C′-stacking now being combined with alternation of flat and corrugated sheets. In **ST2d**, each **hcb**-sheet is corrugated, preserving the AB-stacking of **ST2**.

**Fig. 4:**
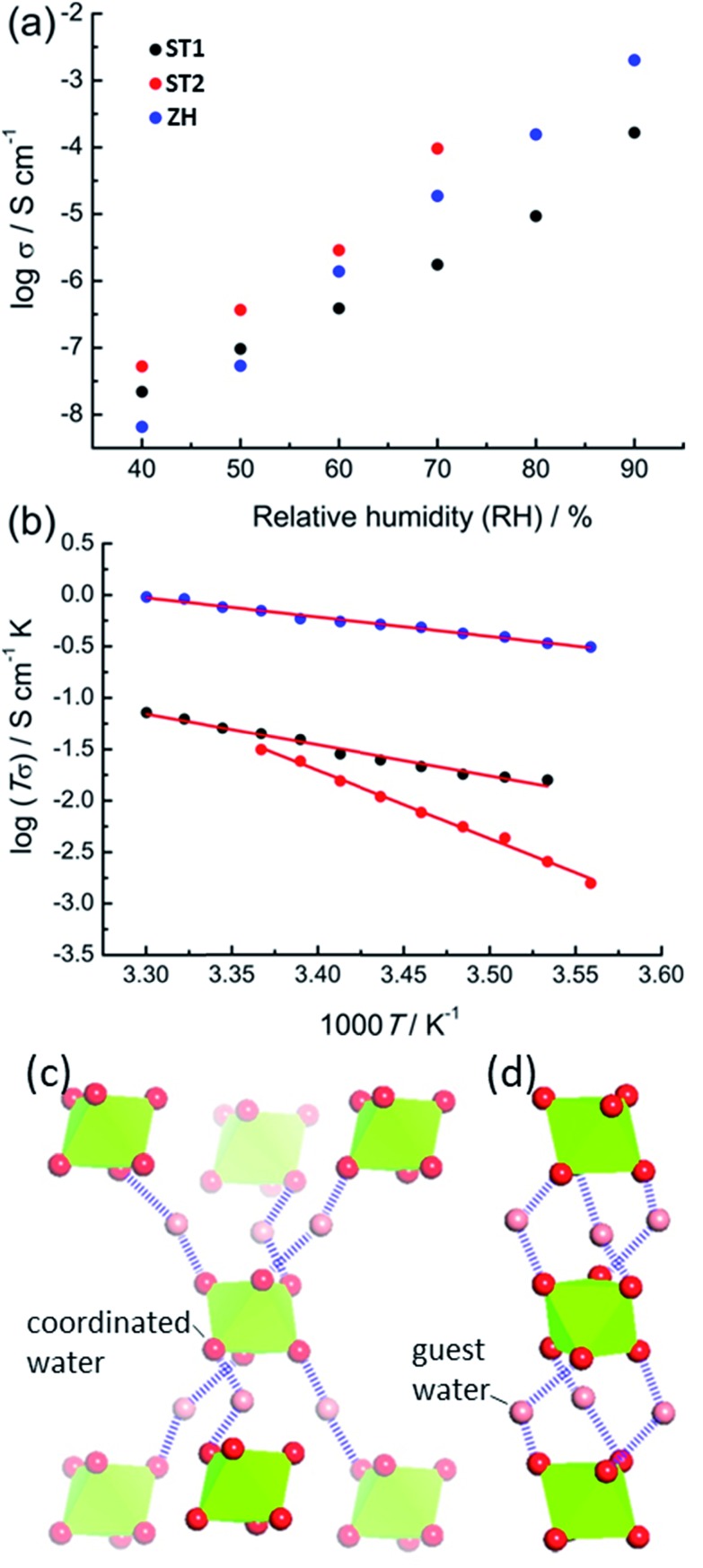
(a) Proton conductivity (*σ*) at 25 °C for synthetic **ST1** (black), **ST2** (red) and **ZH** (blue) at different RH. (b) Arrhenius plots of the proton conductivities for **ST1**(black, at 90% RH), **ST2** (red, at 70% RH), and **ZH** (blue, at 90% RH), with least-squares fits shown as solid lines. Fragments of hydrogen-bonded networks involving guest water molecules and Mg(H_2_O)_6_^2+^ ions in: (c) **ST1** and (d) **ST2**. For clarity, **hcb**-layers are omitted and Mg(H_2_O)_6_^2+^ ions are shown as green octahedra.

The accessibility of bulk synthetic samples for **ST1**, **ST2** and **ZH** enabled the evaluation of suspected proton conductivity by AC impedance measurement on compacted powder pellets. For proton conductivity, all Nyquist plots were obtained under controlled relative humidity (RH) and temperature (see ESI†). Impedance measurements revealed significant enhancement of proton conductivity (*σ*) in **ST1**, **ST2** and **ZH** with relative humidity (Fig. 4a). At 90% RH synthetic **ZH** exhibits a high *σ* of almost 3 × 10^–3^ S cm^–1^, on par with some of the highest room temperature proton conductive MOFs.^27^–^29^ Proton conductivity of synthetic **ST1** is also high, but almost an order of magnitude lower than that of **ZH**. Both **ST1** and **ZH** remained crystalline upon conductivity evaluation, as verified by PXRD patterns recorded after the measurements. Proton conductivity for **ST2** could not be measured above 70% RH, due to deliquescence at higher RH values. However, at all explored RH levels below 70% the conductivity of **ST2** was similar to that of **ZH** and consistently higher than for **ST1**. Indeed, at 70% RH and 25 °C the proton conductivity of **ST2** is among the top values reported for MOFs at modest humidity and room temperature.^30^

The two main mechanisms for proton diffusion are classfied by activation energy (*E*_a_): the vehicle mechanism (*E*_a_ > 0.4 eV) and the Grotthuss mechanism (*E*_a_ < 0.4 eV).^31^,^32^ The activation energies for proton conduction in **ST1** (0.59 eV) and **ZH** (0.37 eV) at 90% RH were established using the Arrhenius method (Fig. 4b). The values are consistent with a mixed Grotthuss–Vehicle proton diffusion mechanism, which puts these materials in the group of superionic conductors, as described by Colomban *et al.*^33^ The *E*_a_ for **ST2** is considerably larger (1.31 eV) compared to **ZH** and **ST1**, due to measurements being performed at 70% RH. In addition, PXRD pattern of **ST2** after conductivity studies at different temperatures revealed partial structural transformation to **ST1**, which could also have affected the high *E*_a_ (see ESI†).

It was reported that high proton conductivity in oxalate MOFs (≥10^–3^ S cm^–1^ at 25 °C, 98% RH) requires the inclusion of additional proton-carrying guests, such as weakly acidic NH_4_^+^ or adipic acid.^14^,^34^,^35^ In such systems, conductivity is strongly influenced by the type and organization of proton-carrying guests.^36^ As the conductivities and activation energies in **ST1**, **ST2** and **ZH** are comparable to such systems, it is likely that Mg(H_2_O)_6_^2+^ ions, in which the proton-donating ability of water molecules is enhanced by coordination to Mg^2+^, act as such protic guests. Differences in *σ* between **ST1**, **ST2** and **ZH** can tentatively be related to different topologies of hydrogen-bonded frameworks formed by Mg(H_2_O)_6_^2+^ ions and water guests (Fig. 4c). As **ST1** and **ST2** have an identical composition, and **ST1** and **ZH** share the same crystallographic structure, it is reasonable to assume that the difference in conduction properties of **ST1** and **ST2** are associated with **ST2** adopting a **ZH**-like hydrogen-bonded guest structure. To verify whether conductivity is due to proton conduction or contributions of other ionic species, additional impedance measurements were performed on deuterated **ST1** and **ZH** at 25 °C and *ca.* 70% saturated D_2_O vapor (see ESI†).^37^ Measurements on both samples revealed a reduction in conductivity upon deuteration, which is consistent with proton conduction. Specifically, measurements on deuterated **ST1** revealed a *ca.* 1.3-fold reduction in conductivity (from 1.76 × 10^–6^ S cm^–1^ to 1.40 × 10^–6^ S cm^–1^) along with a comparable activation energy (0.51 eV). For deuterated **ZH** a more significant 5.8-fold reduction in conductivity was observed (from 1.87 × 10^–5^ S cm^–1^ to 3.22 × 10^–6^ S cm^–1^), along with a small increase in activation energy (0.51 eV, see ESI†).

## Conclusions

In summary, we have shown that MOF minerals zhemchuzhnikovite and stepanovite can exhibit high proton conductivity, enabled by hydrogen-bonding networks involving interstitial water molecules, hydrated metal cation guests and the oxalate based-framework. At the same time, we report the possibility of polymorphism in stepanovite, which enabled observing the effect of hydrogen-bonded framework topology on proton conductivity of an oxalate-based MOF. All of the MOF minerals studied were found to be very stable with respect to the binary oxides, and substitution of iron by aluminum was found to improve the stability of zhemchuzhnikovite. Upon heating, stepanovite polymorphs lose included water guests in a crystal-to-crystal fashion, *i.e.* without disrupting the 2-dimensional open framework layers.

Overall, these results are highly significant in the contexts of geology and materials chemistry, as they demonstrate that naturally occurring MOFs containing small oxalate ligands are thermodynamically stable phases that exhibit functional properties comparable to previously reported advanced materials, including high proton conductivity and framework retention at elevated temperature. We note that this work also presents a simple design for synthesizing high-performing proton conductive materials without sacrificing thermodynamic stability or requiring complex organic components. We are currently investigating the properties of other MOFs based on this design.

## Conflicts of interest

There are no conflicts to declare.

## Supplementary Material

Supplementary information

Crystal structure data
