# Supplementary material for: Functionality in metal–organic framework minerals: proton conductivity, stability and potential for polymorphism
Source: Chem Sci. 2019 Apr 2;10(18):4923–9. doi: 10.1039/c8sc05088k (PMC6510315; doi:10.1039/c8sc05088k)
Supplement: Supplementary file 1 [file SC-010-C8SC05088K-s001.pdf]

Electronic Supplementary Material (ESI) for Chemical Science.
This journal is © The Royal Society of Chemistry 2019

Electronic Supplementary Information

Functionality in Metal-Organic Framwork Minerals: Proton Conductivity, Stability and Potential for Polymorphism

Igor Huskić, Novendra Novendra, Dae-Woon Lim, Filip Topić, Hatem M. Titi, Igor Pekov, Sergey V. Krivovichev, Alexandra Navrotsky*, Hiroshi Kitagawa* and Tomislav Friščić*

Contents

1.	Thermodynamic cycle and data	S2
2.	Hydrogen bonding	S4
3.	Water sorption measurements	S5
4.	Impedance measurements	S6
5.	Selected thermal analysis data	S7
6.	Deuteration of ZH and ST1	S9
7.	References	S10

1. Thermodynamic cycle and data

The calculation of enthalpy of formation ($\Delta H_{f,ox}$ and $\Delta H_{f,el}$) was performed using the following thermodynamic cycles:

Table S1. Thermodynamic cycle used to calculate the formation enthalpy of MOFs from binary oxides ($\Delta H_{f,ox}$).

Reactions	Enthalpy (ΔH, kJ/mol)
$Na^+(aq) + Mg^{2+}(aq) + xFe^{3+}(aq) + (1\text{-}x)Al^{3+}(aq) + 3C_2O_4^{2-}(aq) + 9H_2O(aq) \rightarrow NaMgFe_xAl_{1-x}(C_2O_4)_3{\cdot}9H_2O(s)$	$\Delta H_1 = -\Delta H_{ds}(MOF)$
$NaOH(s) + H^+(aq) \rightarrow Na^+(aq) + H_2O(aq)$	$\Delta H_2 = \Delta H_{ds}(NaOH)$
$0.5Na_2O(s) + 0.5H_2O(l) \rightarrow NaOH(s)$	$\Delta H_3 = \Delta H_{rxn}(Na_2O)$
$MgO(s) + 2H^+(aq) \rightarrow Mg^{2+}(aq) + H_2O(aq)$	$\Delta H_4 = \Delta H_{ds}(MgO)$
$\gamma\text{-}FeOOH(s) + 3H^+ \rightarrow Fe^{3+}(aq) + 2H_2O(aq)$	$\Delta H_5 = \Delta H_{ds}(\gamma\text{-}FeOOH)$
$0.5\alpha\text{-}Fe_2O_3(s) + 0.5H_2O(l) \rightarrow \gamma\text{-}FeOOH(s)$	$\Delta H_6 = \Delta H_{rxn}(Fe_2O_3)$
$Al(OH)_3(s) + 3H^+ \rightarrow Al^{3+}(aq) + 3H_2O(aq)$	$\Delta H_7 = \Delta H_{ds}(Al(OH)_3)$
$0.5\alpha\text{-}Al_2O_3(s) + 1.5H_2O(l) \rightarrow Al(OH)_3(s)$	$\Delta H_8 = \Delta H_{rxn}(Al_2O_3)$
$H_2C_2O_4(s) \rightarrow 2H^+(aq) + C_2O_4^{2-}(aq)$	$\Delta H_9 = \Delta H_{ds}(H_2C_2O_4)$
$H_2O(l) \rightarrow H_2O(aq)$	$\Delta H_{10} = \Delta H_{ds}(H_2O)$
$0.5Na_2O(s) + MgO(s) + 0.5x\ \alpha\text{-}Fe_2O_3(s) + 0.5(1\text{-}x)\ \alpha\text{-}Al_2O_3(s) + 3H_2C_2O_4(s) + (4+x)H_2O(l) \rightarrow NaMgFe_xAl_{(1-x)}(C_2O_4)_3{\cdot}9H_2O(s)$	$\Delta H_{11} = \Delta H_{f,ox}$ (MOF) $= \Delta H_1 + \Delta H_2 + \Delta H_3 + \Delta H_4 + x(\Delta H_5+\Delta H_6) + (1\text{-}x)(\Delta H_7+\Delta H_8) + 3\Delta H_9 + (4+x)\Delta H_{10}$

$\Delta H_{rxn}(Na_2O) = \Delta H_{f,el}(NaOH(s)) - 0.5\Delta H_{f,el}(Na_2O(s)) - 0.5\Delta H_{f,el}(H_2O(l))$

$\Delta H_{rxn}(Fe_2O_3) = \Delta H_{f,el}(\gamma\text{-}FeOOH(s)) - 0.5\Delta H_{f,el}(\alpha\text{-}Fe_2O_3(s)) - 0.5\Delta H_{f,el}(H_2O(l))$

$\Delta H_{rxn}(Al_2O_3) = \Delta H_{f,el}(Al(OH)_3(s)) - 0.5\Delta H_{f,el}(\alpha\text{-}Al_2O_3(s)) - 1.5\Delta H_{f,el}(H_2O(l))$

Table S2. Thermodynamic cycle used to calculate the formation enthalpy of MOFs from the elements ($\Delta H_{f,el}$).

Reactions	Enthalpy (ΔH, kJ/mol)
$0.5Na_2O(s) + MgO(s) + 0.5x\ \alpha\text{-}Fe_2O_3(s) + 0.5(1\text{-}x)\alpha\text{-}Al_2O_3(s) + 3H_2C_2O_4(s) + 6H_2O(l) \rightarrow NaMgFe_xAl_{(1-x)}(C_2O_4)_3{\cdot}9H_2O(s)$	$\Delta H_{11} = \Delta H_{f,ox}(MOF)$
$2Na(s) + 0.5O_2(g) \rightarrow Na_2O(s)$	$\Delta H_{12} = \Delta H_{f,el}\ (Na_2O)$
$Mg(s) + 0.5O_2(g) \rightarrow MgO(s)$	$\Delta H_{13} = \Delta H_{f,el}\ (MgO)$
$2Fe(s) + 1.5O_2(g) \rightarrow \alpha\text{-}Fe_2O_3(s)$	$\Delta H_{14} = \Delta H_{f,el}\ (\alpha\text{-}Fe_2O_3)$
$2Al(s) + 1.5O_2(g) \rightarrow \alpha\text{-}Al_2O_3(s)$	$\Delta H_{15} = \Delta H_{f,el}\ (\alpha\text{-}Al_2O_3)$
$H_2(g) + 2C(s) + 2O_2(g) \rightarrow H_2C_2O_4(s)$	$\Delta H_{16} = \Delta H_{f,el}\ (H_2C_2O_4)$
$H_2(g) + 0.5O_2(g) \rightarrow H_2O(l)$	$\Delta H_{17} = \Delta H_{f,el}\ (H_2O)$
$Na(s) + Mg(s) + xFe(s) + (1\text{-}x)Al(s) + 6C(s) + 9H_2(g) + 10.5O_2(g) \rightarrow NaMgFe_xAl_{(1-x)}(C_2O_4)_3{\cdot}9H_2O(s)$	$\Delta H_{18} = \Delta H_{f,el}\ (MOF) = \Delta H_{11} + 0.5\Delta H_{12} + \Delta H_{13} + 0.5x\Delta H_{14} + 0.5(1\text{-}x)\ \Delta H_{15} + 3\Delta H_{16} + (4+x)\Delta H_{17}$

The measured and calculated enthalpy data are summarized on the tables below.

Table S3. Thermodynamic data measured in 5 molar HCl and calculated data in kJ mol^{-1}

Compound	Formula	ΔH_{ds}	$\Delta H_{f,ox}$	$\Delta H_{f,el}$
**ST1**	$NaMgFe(C_2O_4)_3{\cdot}9H_2O$	91.38±0.66	-422.31±2.29	-5847.41±2.59
**ST2**	$NaMgFe(C_2O_4)_3{\cdot}9H_2O$	92.15±0.47	-423.08±2.24	-5848.19±2.55
**ZH**	$NaMgFe_{0.4}Al_{0.6}(C_2O_4)_3{\cdot}9H_2O$	64.81±1.06	-434.03±3.20	-6113.98±3.45
NaOH	NaOH	-99.45±0.56		-425.93[a]
MgO	MgO	-147.51±0.70		-601.24[a]
Lepidocrocite	γ-FeOOH	-50.76±0.77		-552.00±1.60[a]
Gibbsite	$Al(OH)_3$	-83.48±4.30[a]		-1293.18±1.20 [a]
Oxalic Acid	$C_2H_2O_4$	11.42±0.32		-828.93±0.46 [a]
Water	H_2O	0.5[a]		-285.83 [a]
Sodium oxide	Na_2O			-417.98[a]
Hematite	$\alpha\text{-}Fe_2O_3$			-826.20±1.30[a]
Corundum	$\alpha\text{-}Al_2O_3$			-1675.70±1.30[a]

[a] References[1-5]

2. Hydrogen bonding

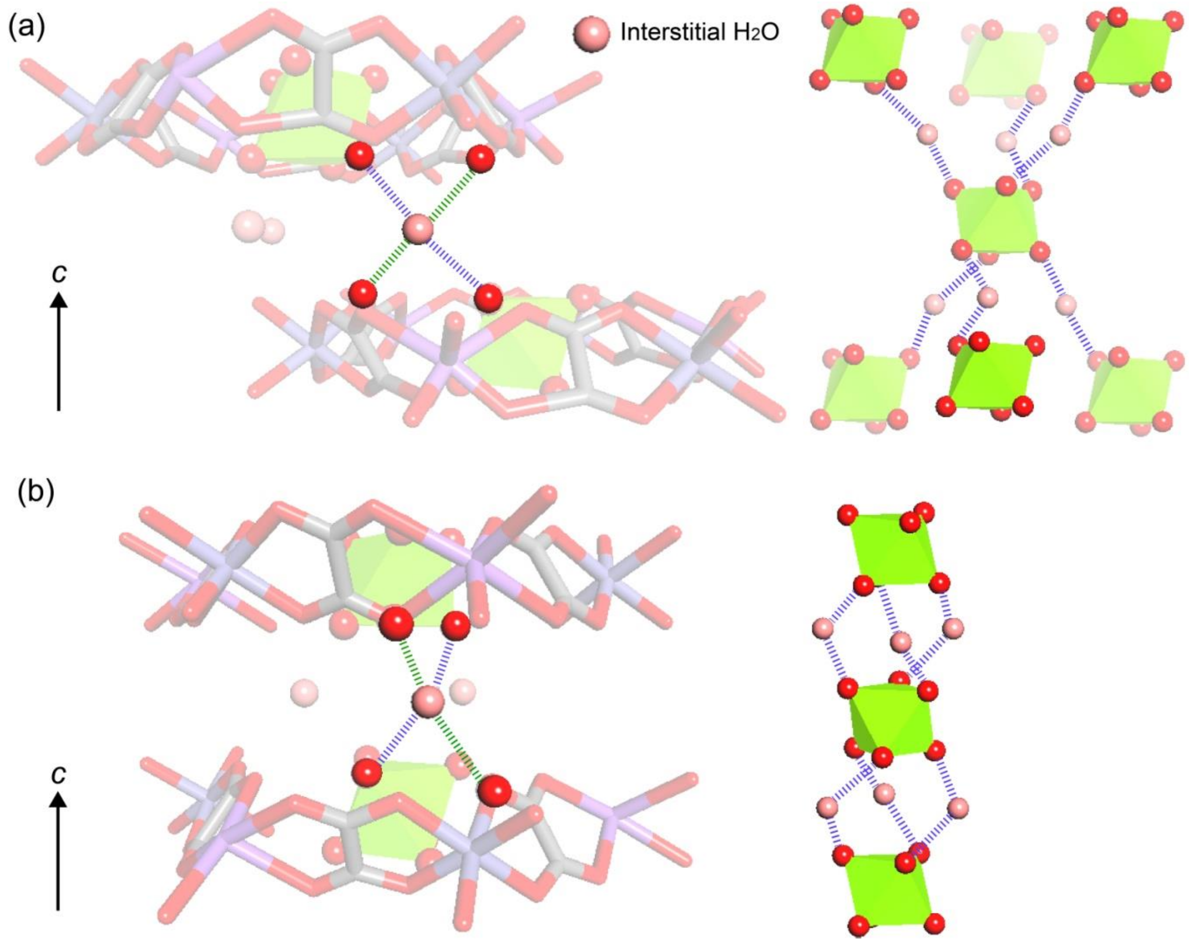

Figure S1. Hydrogen bonding motif of interstitial waters with framework oxygen (green dashed line) and hexaaquamagnesium cation (blue dashed line) in (a) **ST1** and (b) **ST2**. Polyhedra indicate hexaaquamagnesium cation.

3. Water sorption measurements

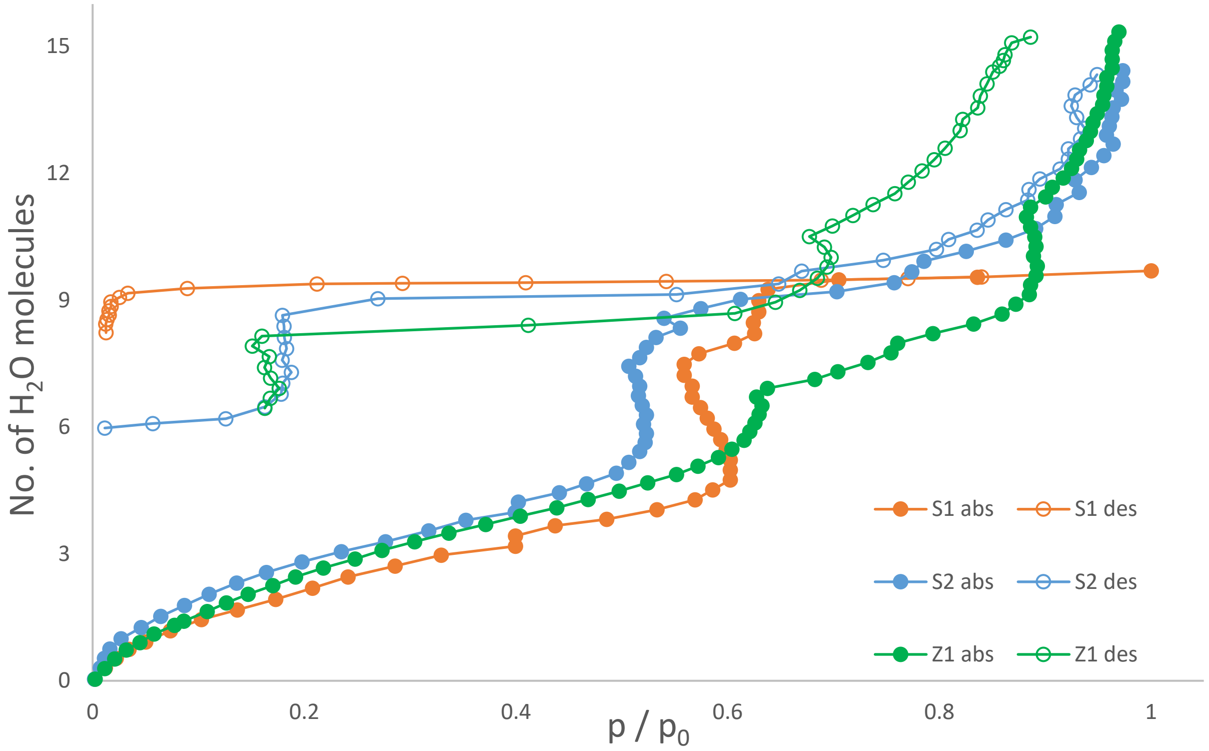

Figure S2. Water sorption isotherms at 298 K of completely dehydrated **ST1**, **ST2** and **ZH**. The filled data points denote the absorption (abs) measurements, while the empty points denote the desorption (des) measurements. The line between data points does not represent fitted values and is included for easier visual recognition.

4. Impedance measurements

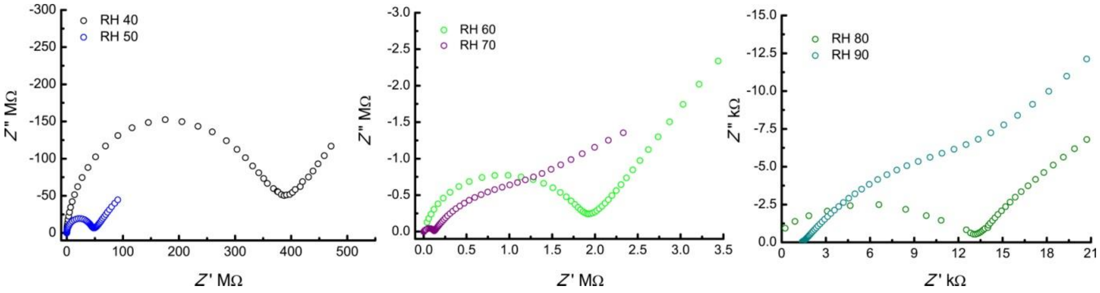

Figure S3. Nyquist plot for **ZH** under the different relative humidity (RH)

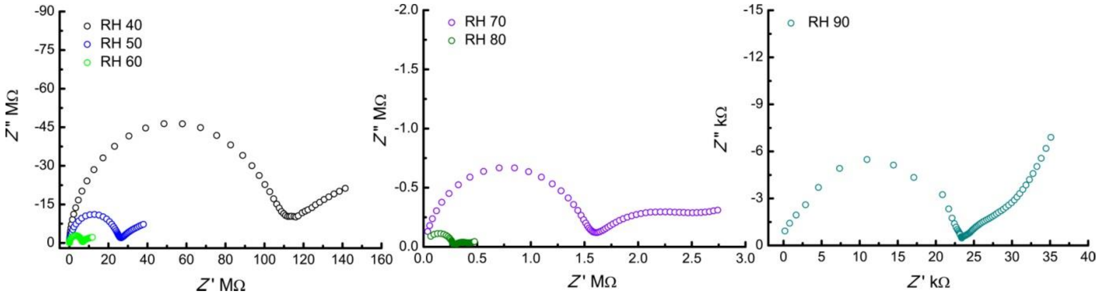

Figure S4. Nyquist plot for **ST1** under the different relative humidity (RH)

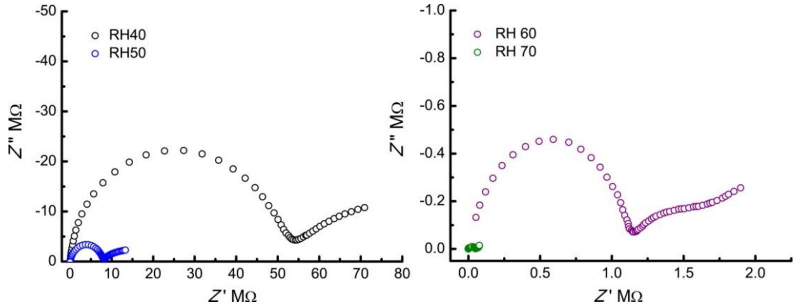

Figure S5. Nyquist plot for **ST2** under the different relative humidity (RH).

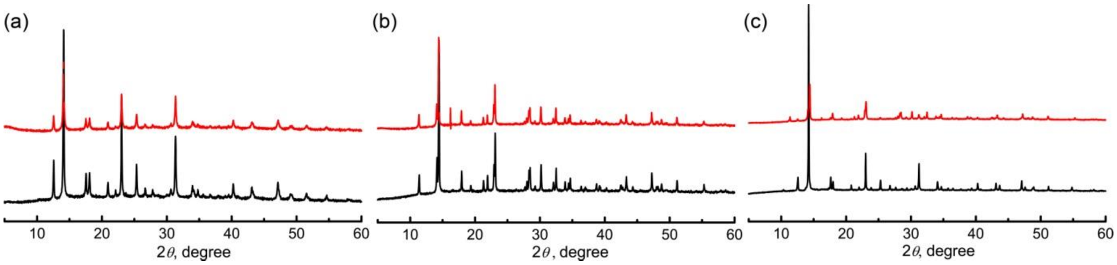

Figure S6. Powder X-ray diffraction patterns before (black) and after (red) impedance measurement for (a) **ZH**, (b) **ST1**, and (c) **ST2**.

5. Selected thermal analysis data

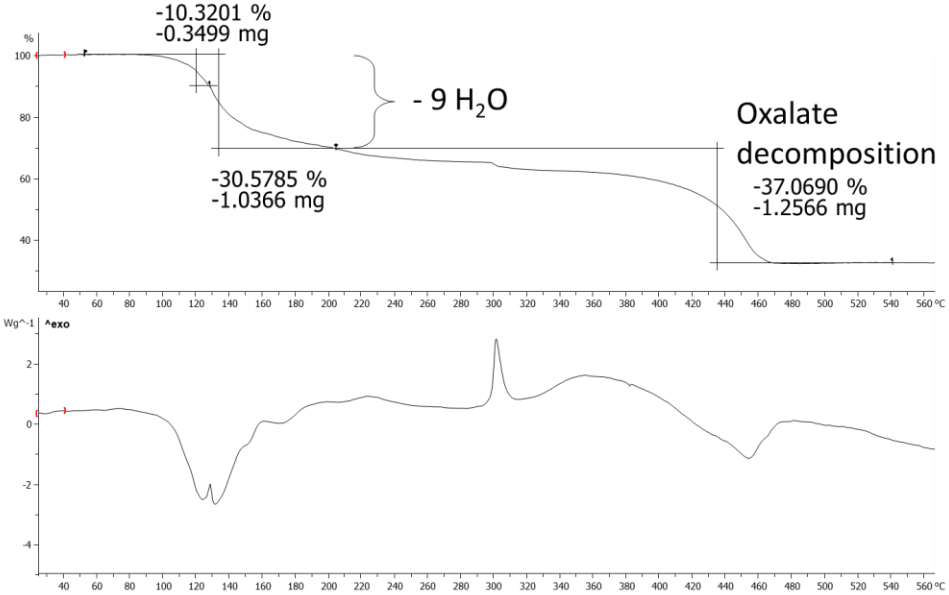

Figure S7. TGA/DSC thermogram of **ST2** measured in a N_2 atmosphere.

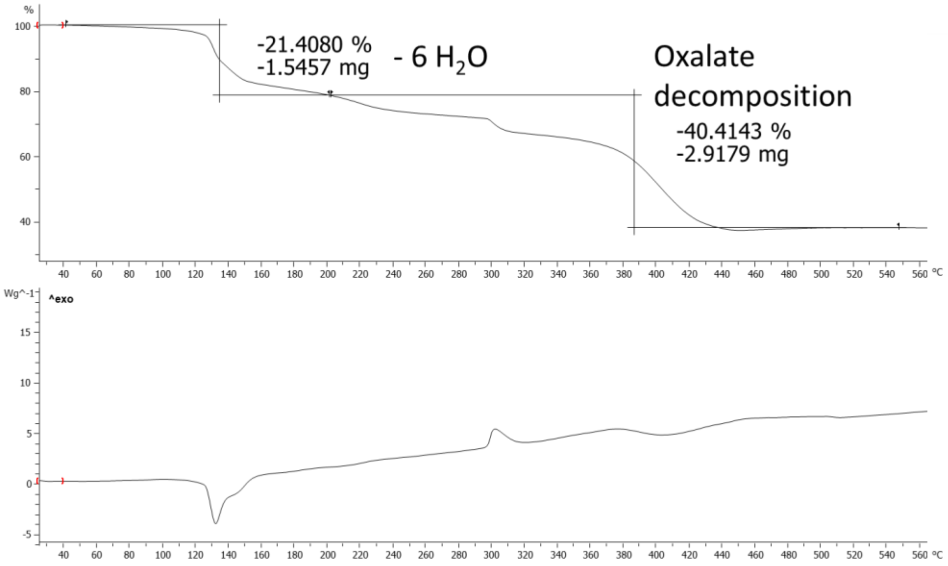

Figure S8. TGA/DSC thermogram of **ST2d** obtained by dehydration of **ST2**, measured in a N_2 atmosphere.

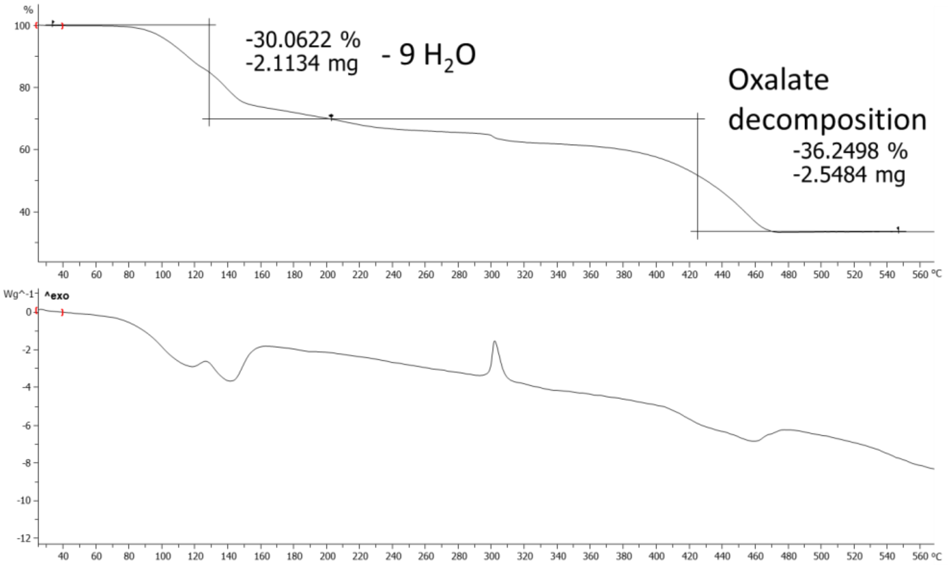

Figure S9. TGA/DSC thermogram of a sample of **ST2d** after exposure to 100% RH, measured in a N_2 atmosphere.

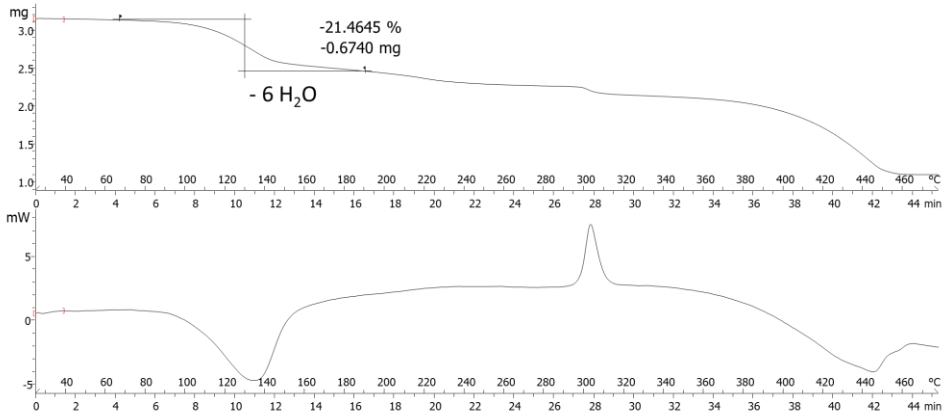

Figure S10. TGA/DSC thermogram of **ST1d**, measured in a N_2 atmosphere.

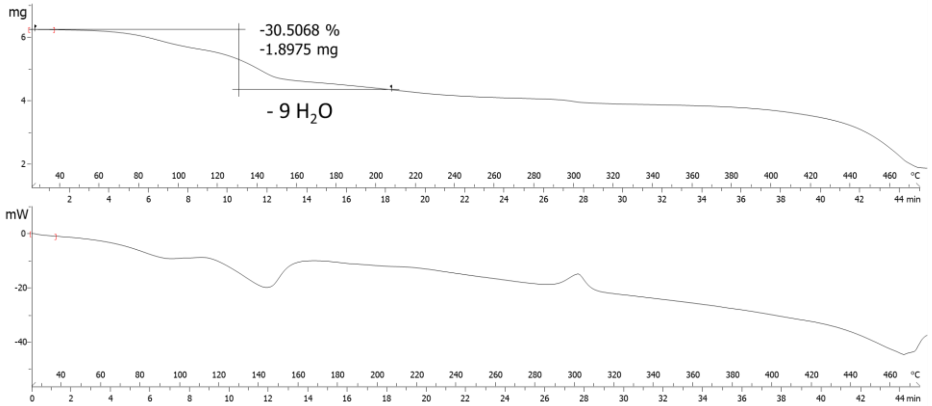

Figure S11. TGA/DSC thermogram of **ST1**, generated by exposing **ST1d** to 100% RH, measured in a N_2 atmosphere.

6. Deuteration of ZH and ST1

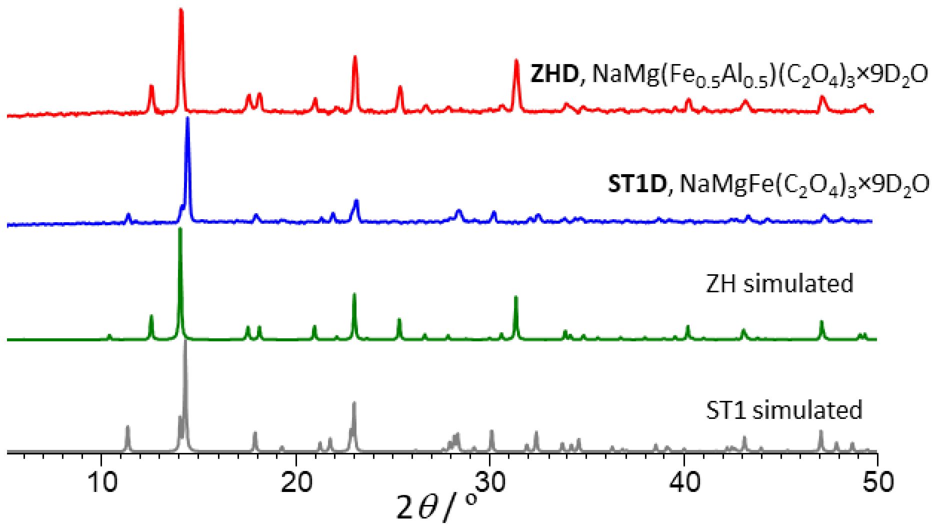

Figure S12. Comparison of PXRD patterns of deuterated samples of ST1 and ZH, compared to simulated patterns for the hydrated parent phases.

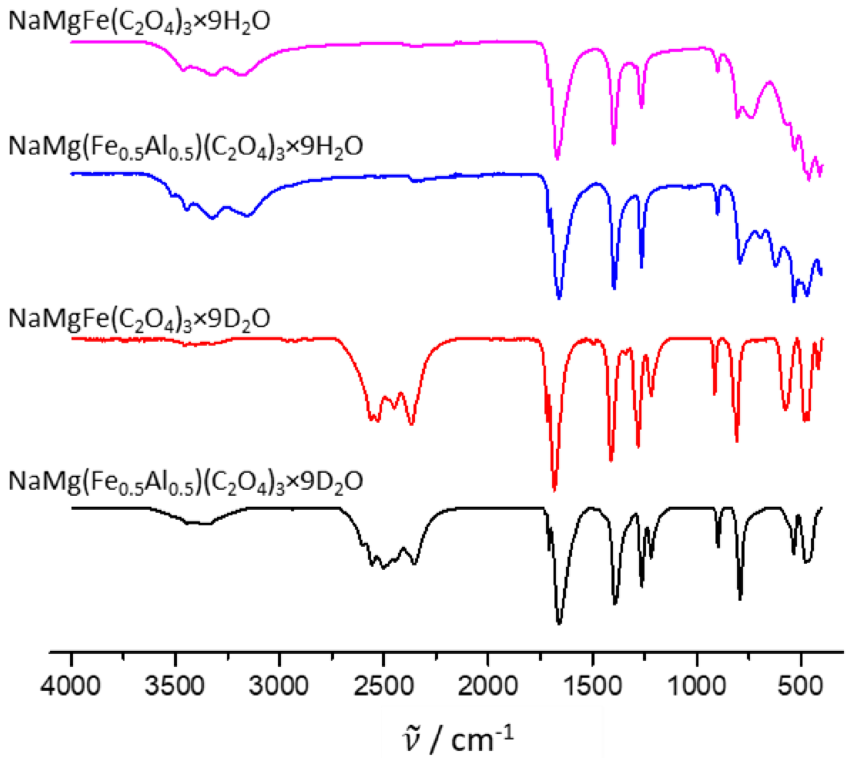

Figure S13. Overlay of FTIR-ATR spectra of deuterated and non-deuterated samples of **ZH** and **ST1**. Replacement of H_2O with D_2O can be seen by the disappearance of bands at ~3300 cm^{-1} corresponding to O–H stretching, and the appearance of bands at ~2500 cm^{-1} corresponding to O–D stretching.

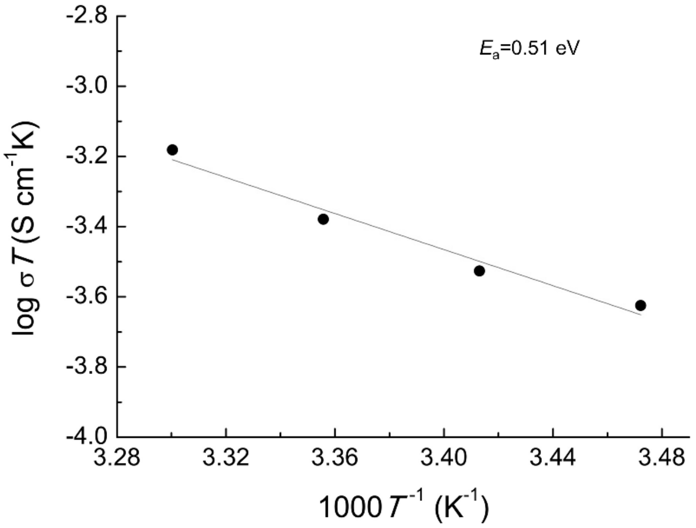

Figure S14. Comparison of proton conductivities for deuterated and non-deuterated sample of **ST1** at ca. 70% saturated D_2O vapor.

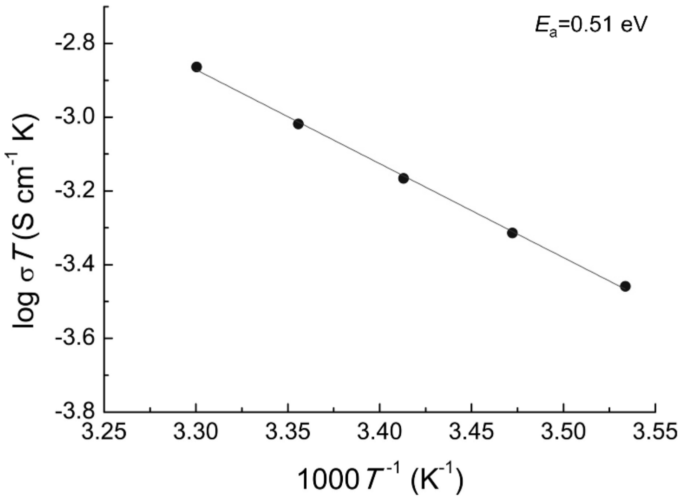

Figure S15. Comparison of proton conductivities for deuterated and non-deuterated sample of **ZH** at ca. 70% saturated D_2O vapor.

7. References

1. C. R. Armstrong, W. H. Casey, A. Navrotsky, *Proc. Natl. Acad. Sci.* **2011**, *108*, 14775–14779.

2. V. B. Parker, *Thermal Properties of Aqueous Uni-Univalent Electrolytes*, Dept. Of Commerce, National Bureau Of Standards, Washington, D.C., U.S., **1965**.

3. J. Malcolm W. Chase, *NIST-JANAF Thermochemical Tables*, Fourth Edition. Washington, DC : American Chemical Society ; New York : American Institute Of Physics For The National Institute Of Standards And Technology, 1998., **1998**.

4. Q. Chen, W. Zeng, *Geochim. Cosmochim. Acta* **1996**, *60*, 1–5.

5. A. Navrotsky, L. Mazeina, J. Majzlan, *Science* **2008**, *319*, 1635–1638.
